# Molecular Imprinting Technology for Determination of Uric Acid

**DOI:** 10.3390/ijms22095032

**Published:** 2021-05-10

**Authors:** Vilma Ratautaite, Urte Samukaite-Bubniene, Deivis Plausinaitis, Raimonda Boguzaite, Domas Balciunas, Almira Ramanaviciene, Grażyna Neunert, Arunas Ramanavicius

**Affiliations:** 1 Department of Functional Materials and Electronics, State Research Institute Centre for Physical Sciences and Technology, Sauletekio Ave. 3, LT-10257 Vilnius, Lithuania; vilma.ratautaite@ftmc.lt (V.R.); urte.samukaite-bubniene@chf.vu.lt (U.S.-B.); b.raimonda@gmail.com (R.B.); 2 Department of Physical Chemistry, Faculty of Chemistry and Geosciences, Vilnius University, Naugarduko Str. 24, LT-03225 Vilnius, Lithuania; deivis.plausinaitis@chf.vu.lt (D.P.); domas.balciunas@chgf.vu.lt (D.B.); 3 NanoTechnas—Nanotechnology and Materials Science Center, Faculty of Chemistry and Geosciences, Vilnius University, Naugarduko Str. 24, LT-03225 Vilnius, Lithuania; almira.ramanaviciene@chf.vu.lt; 4 Department of Physics and Biophysics, Faculty of Food Science and Nutrition, Poznań University of Life Sciences, Wojska Polskiego 38/42, 60-637 Poznań, Poland

**Keywords:** uric acid, molecularly imprinted polymers (MIPs), molecular imprinting technology (MIT), electrochemical methods, conducting polymers, polypyrrole, cyclic voltammetry, pulsed amperometric detection, sensors for biomedical applications, food analysis

## Abstract

The review focuses on the overview of electrochemical sensors based on molecularly imprinted polymers (MIPs) for the determination of uric acid. The importance of robust and precise determination of uric acid is highlighted, a short description of the principles of molecular imprinting technology is presented, and advantages over the others affinity-based analytical methods are discussed. The review is mainly concerned with the electro-analytical methods like cyclic voltammetry, electrochemical impedance spectroscopy, amperometry, etc. Moreover, there are some scattered notes to the other electrochemistry-related analytical methods, which are capable of providing additional information and to solve some challenges that are not achievable using standard electrochemical methods. The significance of these overviewed methods is highlighted. The overview of the research that is employing MIPs imprinted with uric acid is mainly targeted to address these topics: (i) type of polymers, which are used to design uric acid imprint structures; (ii) types of working electrodes and/or other parts of signal transducing systems applied for the registration of analytical signal; (iii) the description of the uric acid extraction procedures applied for the design of final MIP-structure; (iv) advantages and disadvantages of electrochemical methods and other signal transducing methods used for the registration of the analytical signal; (vi) overview of types of interfering molecules, which were analyzed to evaluate the selectivity; (vi) comparison of analytical characteristics such as linear range, limits of detection and quantification, reusability, reproducibility, repeatability, and stability. Some insights in future development of uric acid sensors are discussed in this review.

## 1. Introduction

Uric acid is a biologically active compound, which is found in human fluids (blood and urine). The increased (hyperuricemia) or decreased (hypouricemia) levels of uric acid in the blood and urine are linked to many diseases, including gout, diabetes (type 2), cardiovascular, kidney, and many other [1,2,3,4]. The main source of uric acid in the blood is diets rich in animal proteins (meat, seafood), which are the principal exogenous source of purines. Another dietary source of uric acid is a high intake of fructose [3]. On the other side, hypouricemia is linked to the vegetarian diet lacking purine containing nutrition [5]. Uric acid is also a very important analyte for food chemistry, because high concentrations of this compound show how health is negatively affected by the food, and therefore, the concentration of this compound is especially important for evaluating grain-based products impact on health. The detection of uric acid in food (for example: in wheat, maize, and sorghum grains) may indicate infestation by some insect species [6]. Hence, the importance of the analysis of uric acid is not even debated.

**Figure 1:**
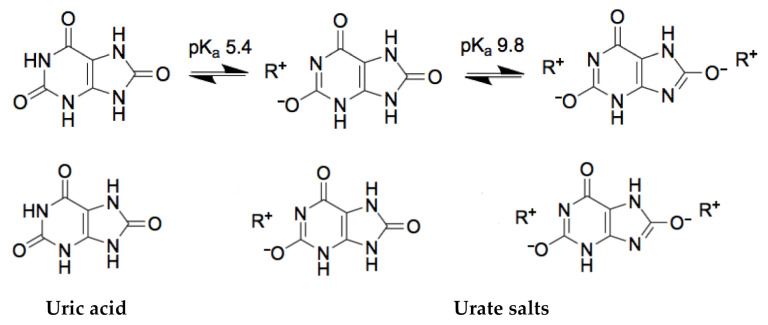
The chemical structures of uric acid and urate-based salts.

From the analytical point of view, there are some important key features: it is the heterocyclic compound, which has two pK_a_ values: pK_a1_ = 5.4 and pK_a2_ = 9.8 (Figure 1) [4]. The human fluids in which uric acid is found are blood and urine. The normal pH value for blood is from 7.35 to 7.4 and for urine pH varies from 5.5 to 7 (an average value is 6.2) [7]. Therefore, the uric acid in human fluids in normal conditions exists as mono-ionic urate ion (from 98% to 99% of all urates) [4]. Estimations of uric acid values are usually based on the measurements of serum uric acid concentration, uric acid clearance, and 24-h urinary uric acid excretion. A normal uric acid level, urinary concentrations, in the urine varies from 250 to 750 mg per 24 h [8].

The solubility of the uric acid in water is 60 mg/L at 20 °C. Meanwhile, the values of uric acid in serum are accepted as normal if it falls between 35 and 72 mg/L in adult males and postmenopausal woman, and between 26 and 60 mg/L in premenopausal women [2]. Although, it is worth mentioning that in the general population, plasma uric acid levels have risen over decades [9]. As it was mentioned before, in blood and urine, uric acid exists majorly as urate, the salt of uric acid. At the elevated concentration of urate in the blood, it tends to form uric acid crystals. Figure 1 represents the chemical conversion of uric acid, which is extremely important for molecular recognition-based systems.

As it is stated previously, uric acid is the electro-active compound. The proposed pathway during electrolytic oxidation of uric acid in acetic acid solution demonstrates that the final products are allantoin (the part of allantoin in total amount final product is 25%), alloxan, and parabanic acid (the part of alloxan and parabanic acid in total amount the final products create each 30%) [10]. The remaining part of the final product of electrolytic oxidation of uric acid in the acetic acid solution is urea. However, the electrochemical oxidation of uric acid in a water-based solution on conductive boron-doped diamond electrode undergoes slightly in a different way. According to the proposed mechanism of electrochemical oxidation in a water-based solution, the final products are urea and alloxan, which are further oxidized to form urea and oxalic acid [11]. From these two examples, it is obvious that the electrochemical oxidation of uric acid depends on the experimental conditions and can still be investigated. Additionally, these two examples clearly demonstrate that the electrochemical oxidation of uric acid is not reversible and should be examined in the certain experimental conditions to clarify the exact oxidation potential. In the previous study of our research group, the electrochemical oxidation potential of uric acid was tested on the 5 MHz gold-coated quartz crystal resonator/electrode in phosphate buffered saline (PBS) solution, pH 7.0, by cyclic voltammetry (CV) and it is found to be approximately equal to +0.6 V [12].

Recently, among many structures exhibiting different affinity towards analyte, artificially made molecularly imprinted polymers (MIPs) are used in analytical systems dedicated for the determination of low [13,14] and high [15,16,17] molecular weight analytes. Some general MIPs designing principles and applications of MIPs in analytical and bioanalytical systems for the determination of both low and high molecular weight compounds are overviewed in recent reviews [18,19,20,21]. Various polymers and polymerization methods are used for the formation of MIPs. The advantage of conducting polymers is that CP-based layers can be deposited using well controllable [22] various electrochemical deposition methods. However, mostly potential cycling, which is followed and controlled by CV [15,23,24], and potential pulses, which are followed by chronoamperometry [16,25,26], are applied. Both the above mentioned methods are providing a great variety of abilities suitable for the most efficient formation and/or modification of formed sensing layers and simultaneous control of formed structure, therefore, the properties of formed CP-based layers can be changed very precisely. Polypyrrole (Ppy) among many other CPs like polyaniline (PANI), polythiophene, poly(ethylenedioxythiophene) (PEDOT), etc., is the most frequently used for the formation of MIP-based sensing structures due to possible electrodeposition from aqueous solutions devices [18,19,22,27]. In addition, the electrochemical methods used for the deposition of CP-based layers enable us to form layers exhibiting very different physical properties. The most interesting properties suitable for selective molecular recognition are provided by the overoxidation phenomenon. Overoxidation of Ppy is rather easily achievable by applying suitable electrode potentials, which remarkably improves selectivity and sensitivity of Ppy-based MIPs devices [18,19,28]. Therefore, overoxidized Ppy-based structures have great properties comparable with that of antibodies or natural receptors and, for this reason, they very often are called artificial antibodies or synthetic receptors, along with some other MIPs that are exhibiting extraordinary selectivity towards imprinted analytes.

Taking into account here mentioned advantages of MIPs, we are aiming to overview: (i) the most suitable polymers and methods recently used for the formation of MIPs applied in sensing systems dedicated for the determination of uric acid; (ii) electrochemical and some other electrochemistry related methods, which are suitable for the determination of analytical signal. Significant attention is also paid to the application of conducting polymers in the design of uric acid imprinted MIPs.

## 2. The Principals of Molecular Imprinting Technology Applied for the Formation of Sensing Platforms

**Figure 2:**
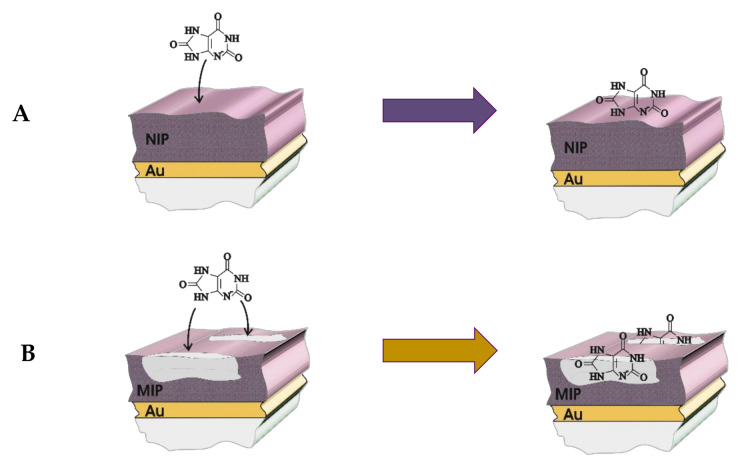
The mechanism of sensing of uric acid on the (**A**) molecularly imprinted polymer (MIP) and (**B**) non-imprinted polymer (NIP) deposited on the gold electrode.

The application of molecular imprinting technology (MIT) is a fascinating way to obtain the highly selective analytical systems. The terminology of MIT is applicable to both (i) inorganic compounds as it was demonstrated on the silica gel-based system at the very beginning of the history of this technology [29] and also for the (ii) polymeric compounds [30]. In case of the MIT application for the systems based on the polymers, the obtained structures are called MIPs. The principal of production of the MIP is based on the artificial modeling of the specific sites for analyte binding on the MIP (Figure 2). The main interaction types of monomer with the template molecule can be distinguished as: chelation, electrostatic, hydrophobic interactions, and the formation of hydrogen bonds [18,19,31]. After removal of the template molecule afterwards, the creation of these specific sites, the three-dimensional cavities are formed. Likewise, the polymers obtained by using a similar procedure as MIP, but without the template molecules are called non-imprinted polymers (NIP) and usually are used for the evaluation and confirmation of MIPs efficiency.

The quality of the MIP is determined by the ability of particular recognition of the analyte despite coexistence of other interfering molecules in the solution in comparison to the NIP. This ability could be characterized by such parameters as imprinting factor (IF), and selectivity factor (α). IF is found according to Equation (1) [29,32]:

(1) $$IF=\frac{Q_{MIP}}{Q_{NIP}}$$

where *Q_MIP_* is the equilibrium binding capacity on the *MIP* and *Q_NIP_* is the equilibrium binding capacity on the *NIP*. Any *IF* value greater than 1 is accepted as the successful outcome of *MIP* formation.

The note about imprinting factor is supported by the findings of the self-assembly of monomers and template molecules [33,34,35]. Such self-assembly involves the ordering of molecules in the pre-polymerization process. Aside from the competing of a favorable and non-favorable energy terms during the ordering of molecules in the self-assembly process, the ratio of the template molecules and the monomers plays an important role on the final efficiency of the MIP. Hence, the increased ratio of the template molecules and the monomers seeking to increase the ability to form the complex of the monomer and template molecule can be the reason of the dropped selectivity.

The parameter α, a selectivity factor, in this case, describes the ability of the MIP to differentiate the analytical signal of analyte from interfering molecule and is found from Equation (2) [29]:

(2) $$\alpha =\frac{Q_{analyte}}{Q_{interfering\;molecule}}$$

where *Q*_(*analyte*)_ is the equilibrium binding capacity of analyte and *Q*_(*interfering molecule*)_ is the equilibrium binding capacity of the interfering molecule on the MIP.

The equations involving *Q*, as it was suggested by Ndunda [29], is more common for chromatographic evaluation methods of MIPs and NIPs. In case of MIP application for the electrochemical analysis methods, the evaluation of *Q* as the equilibrium binding capacity becomes complicated. *Q*, an adsorption capacity (mg/g of polymer), refers to the amount of the template molecule bound to the polymer at the equilibrium.

(3) $$Q=\frac{(C_{0}-C_{x})\times V}{m}$$

where *C*_0_ (mg/L) is the concentration of analyte before adsorption, *C_x_* is the concentration of analyte after adsorption, *V* (L) is the volume of one mole of analyte, and m (g) is the mass of the MIP used for experiment.

To avoid difficult *Q* evaluations, *Q*_max_ as the maximum binding amount is usually used.

Adsorption, desorption, diffusion of analyte, charge transport, and charge transfer are the key aspects at which affect the performance of MIP [36]. Sharma et al. [36] as well as the first scientific report mentioning this effect published by Piletsky et al. [37] links the mechanism of the MIP working principles to the ‘gate effect’. The generalized meaning of the ‘gate effect’ primarily and directly is related to the phenomenon of electrode (modification of electrode) and electrolyte interaction [36]. Moreover, one limitation of four of the MIP technologies regarding to commercialization, as it was stated by Piletsky and Turner [38], is related to ‘the need for a substantial increase in polymer affinity and the improvement of the ratio between specific and nonspecific binding’.

Despite other advantages of electrochemical polymerization, such as the control of the thickness of the polymer layer and the ability of polymer formation on any shape of electrode surface, electrochemical polymerization, and MIP formation method can be acknowledged as meeting the green chemistry requirements [39].

MIPs in analytical systems can displace some biomolecules and in this way can resolve several important aspects within overcoming such drawbacks as: (i) poor stability [40,41]—under harsh conditions like high temperature, wide pH range, organic-based solvents, biomaterials are not suitable and, therefore, under such conditions bioanalytical systems are facing problems related to proper operation, although study [40] of theophylline imprinted methacrylic acid–ethylene glycol dimethacrylate co-polymer revealed that this co-polymer withstand exposure to temperatures of up to 150 °C (for 24 h) without the loss of affinity for the template. The polymers exhibited remarkable resistance to extremes of pH, organic base, to autoclave treatment and demonstrated a long-term stability [41]; (ii) a study [42] has demonstrated multiple reuse of molecular templates in the formation of imprinted polymers (more than 30 times without the loss of performance) through the use of an immobilized MIP-based nanoparticles (NPs); (iii) high costs—compared to other recognition systems, MIPs offer low-cost technologies [43]; (iv) time-consuming production, meaning grinding and sieving of materials in bulk polymerization case. MIPs overcome this aspect with suspensions, co-precipitations that are simpler because one-step polymerization is used; (v) complicated quality control, limitations in small molecule recognition (signals depend on the molecular weight); (vi) MIP compositions often have to be optimized in a bulk state (this may lead to differences in adhesion properties between the bulk and the sensor). The performance of MIPs has also been enhanced by incorporating nanomaterials, metalo-organic frameworks, magnetic and others NPs or using various MIT–surface imprinting, segment imprinting, multi-responsive imprinting etc.

Finally, electrochemical methods employing MIP can meet some other advantages or limitations: covalent binding is less flexible since reversible reactions are limited and thermodynamic equilibrium is difficult to reach, because the binding and dissociation of imprinted analyte are rather slow [44]. Non-covalent binding without disruption of the interactions is used for relatively rapid analyte binding and removal [24,26,44]. A regeneration is required for sensors with integrated metal ions or metal complexes [44]. MIPs ability for the suppression of interferences in the electrochemical applications by acting as shape-selective filters can be assigned as a key advantage [45]. The thickness of the MIP layer is important parameter of MIP-based analytical systems. The thickness of the MIP layer can be easily adapted and synthesized by selection of appropriate electrochemical parameters. A thicker film afforded more imprinted cavities, which benefits the recognition of target molecules. However, if the MIP film is too thick, some of the imprinted cavities could be buried rather deeply in the polymer matrix, and therefore, the target molecules are difficult to access the active site due to the high mass-transfer resistance. On the other side, the thinner MIP film produces rather limited amount of imprinted cavities, and in such a way target molecule recognition is complicated [46]. It is important to note that the same publication information defines the ideal thickness of polymeric layer for effective analytical signal transduction, which is in the range from 10 to 100 nm [30].

For the application of the MIPs in the design of electrochemical sensors, the most suitable electrochemical analytical signal determination methods should be selected. One of the best systematic analysis of methods used in electrochemical sensing has been presented by Blanco-Lopez et al. [30]. According to this classification, the main methods are: amperometric, voltammetric, conductometric, potentiometric, capacitive, and ion-sensitive field-effect transistors (ISFET) [21,30,31].

For the examination of the electrode–electrolyte interface, electrochemical impedance spectroscopy (EIS) measurements are used and Randles–Ershler equivalent circuit is the most often applied for the interpretation of such interfaces [36]. The main parameters of the Randles–Ershler equivalent circuit include resistance of solution (R_s_), resistance of charge transfer (R_ct_), the Warburg impedance (Z_w_), and the capacitance of double-layer (C_dl_) of the electrode. The most often used ways of plotting the impedance spectra are Nyquist and Bode plots [47]. The Nyquist plots are preferable for impedance plotting due to the ability to predict the elements of the circuit and it is usually used for studying electron-transfer kinetics. From the Bode plots, it can be evaluated how the frequency depends on impedance. However, these two ways of plotting impedance do not expose the impedance and capacitance relationship. This gap (shortage) can be easily bypassed by plotting the impedance in Z_real_–C_real_ coordinates [48].

Division of the EIS sensors into two groups (Faradaic and non-Faradaic) is supported by the presence of the redox species in the interface of the electrolyte and electrode due to which electrical charge transfer is observed [49]. As it was stated of the same above cited study, higher sensitivity is more characteristic for faradaic sensors neither for non-faradaic only due to the current of redox reactions. EIS, besides its simplicity, provides a characterization of each step of the MIP synthesis and highly sensitive readout of target rebinding. EIS monitoring is based on the theory of the electric double-layer. Note: A system containing minute quantity of redox-active material may lead to an error in determination, therefore pseudo-capacitance effects of the Faradaic process should be also taken into account [50].

Taking everything into account, the quality of the MIPs is determined by the ability of a unique recognition of the analyte despite the coexistence of other interfering molecules and other species in the solution, association and dissociation of the imprinted analyte, and the method of interpretation of various interfaces.

## 3. The Main Interfering Molecules for Uric Acid Determination

The meaning of the notion ’interference’ is the act of interfering, invading, or poaching by molecules different from the analyte. In chemical analysis, the process of interference is understood as the combination of the analytical signal of several compounds at which the resulting signal is the superposition of the separately measured signals. In this case, the signal of the compound of interest cannot be qualitatively and quantitatively identified and analyzed. As it is obvious from the description mentioned above, the analytical method has a significant role on the characterization of the interfering molecules. In the case of the analysis by various electrochemical methods, the key feature of the molecules of interest and the interfering molecules are the oxidation potential. Therefore, the aim of the development and optimization of electrochemical method is usually the alternation of such analytical conditions that force the shift of oxidation potentials of the molecules of interest and the interfering molecules qualitative and quantitative analysis are possible. In the case of microgravimetric analysis of the sample, the interfering molecules do not correspond to the found in the electrochemical methods, e.g., CV due to the principal differences of analytical methods. The transport of charge across the interface between an electrolyte and an electrode is affected by various processes and factors, which are the research object of electrochemical methods, while the research object of quartz crystal microbalance (QCM) is the measurement of a mass variation per unit area by measuring the change in frequency of a quartz crystal resonator. Sometimes a reasonable combination of QCM and electrochemical methods can be applied for simultaneous determination of both signals and this combination is called the electrochemical quartz crystal microbalance (EQCM) [12,25,48,51].

As it was mentioned above, uric acid is an electro-active compound that has oxidation potential close to +0.6 (±0.1 V) V vs. Ag/AgCl [10,12,52]. Electrochemical methods were used only for uric acid or for simultaneous determination of several target molecules including uric acid in previous studies [53,54,55,56,57,58]. For the discussion of interfering molecules, it should be noted that the primarily predicted purpose of the sensor, for example, for dopamine or ascorbic acid [59,60] or any other target molecule detection, in most cases, means the optimized conditions at which the molecules are identified as interfering and analyzed at the optimized conditions of analysis. Therefore, the terms mentioned in the articles listed below are optimized for primarily of other target molecules, but the conditions of analysis of uric acid are qualitative and quantitative. This principle is rather suitable if the electrochemical system is not discriminating the oxidation of analytes on the electrode. Such examples of electrochemical analysis are when the electrode is modified with Ppy, reduced graphene oxide (RGO), or graphene oxide (GO): ZnO–Cu_x_O/Ppy for the determination of ascorbic acid, dopamine, and uric acid [61,62], Cu_x_O-ZnO/Ppy/RGO for the determination of ascorbic acid, dopamine, uric acid, and folic acid [56], or polytetraphenylporphyrin/Ppy/GO for uric acid detection [62,63].

A different case is when the electrode is modified with MIP. The signal of electro-oxidation of interfering molecules usually is significantly lower on MIP than that of the target molecule. Such examples are when uric acid is determined on the MIP Ppy modified electrode with dopamine imprints [64], with ephedrine imprints [65], with ascorbic acid imprints [59], and MIP polyaniline modified electrode with ascorbic acid imprints [66] or piezoelectric TiO_2_ with uric acid imprints [67].

In the case of the MIP developed for uric acid determination, the electrochemical deposition of Ppy was performed together with uric acid templates and the obtained film was characterized with significantly higher sensitivity for uric acid than that one for interfering molecules i.e., caffeine and glucose [12]. The mentioned determination of uric acid was performed on gold-coated quartz crystal resonator used in QCM system.

Similar effect was described in the case of the formation of the uric acid imprints in TiO_2_ [51]. The MIT modified TiO_2_ with uric acid imprints was used in piezoelectric measurements and exhibited significantly higher sensitivity to uric acid than that for interfering molecules such as ascorbic acid, urea, glucose, glutamic acid, purine, or cytosine. The linear range of uric acid detection was from 0.04 to 45 μM and the detection limit 0.01 μM in the mentioned MIT TiO_2_. The formation of TiO_2_-based structures seems especially promising MIT direction due to the great variety of titanium oxide forms, that can rather easily be converted into another one [68] and exhibit well-detectable electrical, electrochemical, and optical properties that are strongly affected by adsorption/desorption of various chemical compounds. Therefore, TiO_2_-based MITs seem rather promising for the development of various sensors [69].

## 4. Molecular Imprinting Technologies for Determination of Uric Acid

Electrochemical approaches have been adapted for the readout of MIP or MIT sensors, which were appropriate for the uric acid measurements. These main approaches include (i) faradaic and non-faradaic current measurements; (ii) detection of generated redox-active products; (iii) detection of the signal, which is generated by the binding of the target molecule.

In 2016, Tang et al. [70] developed an MIP sensor for the determination of uric acid in physiological liquids. A photoresponsive surface molecularly imprinted polymer (SMIP) on ZnO nanorods was fabricated. The performance of the MIP by means of photoresponsive properties was evaluated, high recognition ability, and fast binding kinetics toward uric acid. The sensor showed a dissociation constant of 32.2 μM in aqueous NaH_2_PO_4_ buffer at pH = 7.0 and a maximal adsorption capacity of 1.45 μmol/g. They have shown that the binding kinetics of uric acid on ZnO-SMIP reached the binding capacity of about 32.0%, and the equilibrium was achieved in 3 h. The absorption efficiency was calculated to be 32.0%, 9.8%, and 8.5% of the adsorption capacities of uric acid, adenine, and guanine, respectively. They revealed that SMIPs exhibit the advantages of uniform morphology, high surface to volume ratio, more recognition sites and easy preparation process, complete removal of templates, better accessibility to the target species, and faster binding kinetics [70]. Structural analogs (adenine, guanine) were used as competing compounds to estimate the selectivity of ZnO-SMIP toward uric acid. It is remarkable that ZnO-SMIP can be also imprinted by larger molecular weight analytes (such as) and successfully applied in the design of sensors [71].

MIP based on a novel monomer of 2-amino-5-mercapto-1, 3, 4-thiadiazole (AMT) and RGO composite was developed in 2018 by Zeng et al. [24]. This great study was performed on the determination of two analytes, uric acid and tyrosine, simultaneously. The electrochemical sensor was prepared exploiting MIP and RGO composite—the MIP layer was constructed on the well-dispersed RGO nanosheets using a facile electropolymerization method. A glassy carbon electrode was chosen for MIP/RGO deposition by CV. After polymerization, the uric acid and tyrosine were extracted by washing in ethanol for 30 min. As authors stated the recognition of uric acid and tyrosine depend on interaction of hydrogen bonding and π-π stacking. The uric acid at pH 5.0 exists primarily as an enol form. Hence, the hydrogen group in the enol form of uric acid can serve as hydrogen bonding donor and interact with the nitrogen in the poly-AMT-based MIP layer. The obtained films were characterized by several methods—CV, EIS, and differential pulse voltammetry (DPV) in PBS solution, pH 5.0, with a redox probe of 10 mM K_3_[Fe(CN)_6_]/K_4_[Fe(CN)_6_]. The sensor showed wide linear ranges for uric acid (0.01 μM–100 μM) and tyrosine (0.1 μM–400 μM) with detection limits of 0.0032 μM and 0.046 μM, respectively. The selectivity was evaluated by testing the DPV responses of 0.4 μM tyrosine and 0.4 μM uric acid in the presence of 50-fold higher concentration of interfering compounds, including dopamine, epinephrine, adenine, xanthine, ascorbic acid, and glucose. Repeatability was investigated by detecting 0.4 μM of tyrosine and 0.4 μM of uric acid for 11 times using the same MIP/RGO sensor. Satisfying repeatability was obtained with the relative standard deviation (RSD) values of 3.86% and 4.23%. The reproducibility was evaluated by detecting 0.4 μM of tyrosine and 0.4 μM of uric acid using 6 different MIP/RGO sensors. The RSD value of 4.68% was obtained. The stability of the MIP/RGO sensor was evaluated by testing the DPV response after the sensor was storing for 20 days at room temperature. The sensor retained 90.6% of the initial response, which indicated a good stability [24]. Noteworthy, the study declares the optimum monomer/template ratio of 20:1.

In 2020, another electrochemical sensor based on dual-template MIP with nanoporous gold leaf (NPGL) was established for the simultaneous determination of dopamine and uric acid by Li et al. [23]. Under the optimal conditions, the sensor showed a good linear range of 2.0–180 μM for dopamine at a working potential of 0.15 V (vs Ag/AgCl) and 5.0–160 μM for uric acid at 0.35 V (vs Ag/AgCl), with the respective detection limit of 0.3 and 0.4 μM (S/N = 3). The responses maintained higher than 96% of the initial values after 30-day storage, and the day-to-day relative standard deviation was less than 3.0%. Real sample simultaneous determination of dopamine and uric acid was conducted within bovine serum. Selectivity dual-template MIP sensor for dopamine and uric acid was investigated by recording sensor responses to exposure to structural analogues of dopamine (3,4-dihydroxyphenylacetic acid, epinephrine, norepinephrine) and other potential interferences (urea, ascorbic acid, glucose, NaCl, MgSO_4_, and folic acid). The reusability of this sensor was explored by detecting dopamine and uric acid at five different concentrations using the same dual-template sensor. The sensor showed satisfactory reusability with a relative standard deviation (RSD) of 2.87% for dopamine and 2.18% for uric acid. Furthermore, the reproducibility of this sensor was evaluated using six independent dual-template MIPs by measuring the peak currents of 10 μM of dopamine and 70 μM of uric acid. The RSD of dopamine and uric acid were 3.12% and 2.82%, respectively, indicating good reproducibility of dual-template. In order to investigate the repeatability, the sensing system was used to perform 20 repetitive measurements in PBS solution containing 10 μM of dopamine and 70 μM of uric acid through a rebinding-extraction process. The RSD were 2.59% and 2.04% for dopamine and uric acid, respectively, exhibiting remarkable repeatability.

Nanoparticles exhibit exceptional and different properties compared with macro- ones and in such a way, MIPs characteristics can be enhanced or new functionalities can be obtained when NPs are incorporated in the polymeric structure. In the work by Mujahid [72], molecularly imprinted titania NPs (MINPs) of 100–150 nm were developed for the recognition of uric acid. They have carried out selectivity experiments by immersing MINPs in ascorbic acid and guanine solutions of equi-molar concentration. MINPs showed higher binding affinity toward uric acid as compared to ascorbic acid and guanine. The characteristics of uric acid imprinted titania NPs such as selectivity and their binding capacity after complete template removal in the process of washing were studied by UV–vis spectrophotometric analysis. The results of static adsorption capacity suggested that the binding capability of MINPs increases linearly with the increasing concentration of uric acid and the tested concentration range is comparable with the normal to elevated levels of uric acid in blood serum, in the range of 10–95 μM. The non-imprinted NPs (NINPs) were considered as control (reference) as they showed negligible effect in comparison to MINPs.

A similar study, but with urea instead of uric acid, was published by Yang et al. in 2015 [67]. They developed a nonenzymatic piezoelectric sensor for urea detection [67]. The sensor exhibited high sensitivity in urea detection, with a linear range from 0.04 to 120 μM and a limit of detection of 0.01 μM. Moreover, the sensor presented outstanding selectivity while used in coexisting systems containing various interfering molecules with high concentration. The dynamic range of the urea sensor was studied by monitoring the urea solution in the concentration range from 0.01 to 150 µM. The analytical application of the urea sensor confirmed the feasibility of urea detection in the urine sample. The reproducibility of the sensor was evaluated by measuring the response signal 20 times. The obtained results indicated that the RSD of frequency response was 1.31%. The long-term stability revealed that after storage of 5 months at room temperature, the frequency change decreased by only 2.07% compared to the initial frequency response. The sensor exhibited a rapid response—less than 2 s.

A piezoelectric sensor was established for the uric acid detection [51]. A surface modification and MIT were combined. Such system was based on the immobilization of TiO_2_ NPs on EQCM electrode. The TiO_2_ NPs on QCM electrode were obtained by the sol-gel method. Sensitivity with linear detection rate from 0.04 to 45 μM was detected and a limit of detection was 0.01 μM. The obtained recovery determination of uric acid in the urine sample reached 96.67% to 101.50%. The reusability was evaluated by measuring the response signal 15 times with the decrease of a signal by only 1.8% at the last cycle. In addition, sensors stability was tested within a 6 months period—the sensor retained above 96% of its original frequency response, demonstrating high stability.

In the case of molecular imprinting technology application on Ppy, there is one feature that is assumed as more disturbing for analysis neither amend it. This is the overoxidation of the Ppy at the positive potential over 1 V [73]. During overoxidation of Ppy, the conjugated backbone of the polymer becomes ‘damaged’, therefore, the polymer loses its conductivity and becomes an insulator.

An amine–imide (poly(PD–BCD)) imprinted with uric acid was deposited on indium–tin oxide (ITO) electrode [46]. Poly(PD–BCD) imprinted with uric acid was prepared by admixing of poly(PD–BCD) at various concentrations with uric acid dissolved in 1-methyl-2-pyrrolidinone. The obtained mixture was dropped on the ITO electrode and dried at the elevated temperature. Template molecules extracted by washing with water and electrochemically by potential cycling from 0.1 to 0.9 V at a scan rate of 100 mV/s in deionized water. The films were characterized by CV, linear sweep voltammetry (LSV), and amperometry. Only ascorbic acid was evaluated as the interfering molecule. Despite the use of a light-transmitting electrode, its potential has not been fairly exploited.

Graphene doped molecularly imprinted electrochemical sensor for uric acid was developed [26]. For this purpose, two MIPs deposited on the glassy carbon electrodes were compared. First was graphene doped chitosan MIP and the second was chitosan MIP. The MIPs were obtained amperometrically under potential of −1.1 V which was applied for 3 min. All MIPs were prepared at constant uric acid and chitosan concentrations and different concentrations of graphene. The uric acid from obtained MIPs was extracted by potential cycling at the scan rate of 800 mV/s and a potential range from 1.5 to −1.0 V for 40 cycles in 0.1 M PBS. H-bond interaction between the chitosan and uric acid was proven by FTIR, meanwhile electrochemical behaviors of uric acid were evaluated by CV, EIS, and chronocoulometry. Furthermore, the reproducibility of this sensor was evaluated using six independent dual-template MIPs by measuring the peak currents of 10 μM of dopamine and 70 μM of uric acid. The RSD of dopamine and uric acid were 3.12% and 2.82%, respectively, indicating good reproducibility of dual-template. In order to investigate the repeatability, the sensing system was used to perform 20 repetitive measurements in PBS solution containing 10 μM of dopamine and 70 μM of uric acid through a rebinding-extraction process. The RSD were 2.59% and 2.04% for dopamine and uric acid, respectively, exhibiting remarkable repeatability in presence of redox probe K_3_[Fe(CN)_6_]/K_4_[Fe(CN)_6_]. The selectivity of the graphene doped chitosan MIP was proved in the presence of such interfering molecules like ascorbic acid, dopamine, urea, caffeine, and xanthine. The key conclusion of this study goes to the influence of graphene quantity on the oxidation peak current. In the study, it was found that at the correct concentration of graphene oxidation peak current tends to increase. The observed phenomenon was explained by high specific surface area and excellent conductivity of the graphene, which enlarge the effective adsorption area of the GR-CS-MIP sensor and accelerate the charge-transfer between the sensor and solution.

MIP based on molecularly imprinted poly-methacrylic acid on multiwalled carbon nanotubes (MIP-MWCNTs/PMAA) was obtained by a thermal polymerization method initiated by 2,2′-azobis(2-isobutyro) nitrile (AIBN) [74]. The uric acid from MIP-MWCNTs/PMAA particles was extracted with water:methanol (3:1, *v*/*v*) mixture. Such thermally polymerized MIP-MWCNTs/PMAA was deposited on glassy carbon electrode and used for amperometric, CV, and LSV measurements in 20 mM PBS solution, pH 7.4. The results of the study prove that MIP-MWCNTs/PMAA is about 4.4 times more efficient than neither NIP-MWCNTs/PMAA.

The carbon paste electrode was modified with MIPs for voltammetric determination of uric acid [75]. The thermal polymerization of acrylic acid (AA) and ethylene glycol dimethacrylate (EGDMA) in presence of uric acid was initiated by 2,2′- azobis(2-isobutyro) nitrile (AIBN). The uric acid from the obtained MIP was extracted by washing the MIP particles successively for nine times using 50 mL 0.1 N HCl and ethanol mixture (1:1 *v*/*v*) with stirring for 4 h. The hydrogen bond formation between UA and AA was confirmed by Fourier transform infrared (FTIR) spectra. The MIPs were characterized by CV, differential pulse adsorptive stripping voltammetry (DPAdSV), EIS, and chronoamperometry in PBS solution of pH 7.0. Glucose, lactose, glycine, tryptophan, and ascorbic acid were evaluated as interfering molecules.

A sensor with molecularly imprinted polydopamine modified with nickel NPs wrapped with carbon (Ni@BC-MIP) for electrochemical detection of uric acid was developed [14]. The glassy carbon electrode first of all was coated with carbon-enwrapped nickel NPs, later it was coated with polydopamine imprinted with uric acid by CV. The template of the Ni@BC-MIP just after electrochemical polymerization was extracted with methanol mixed with acetic acid (9:1, *v*/*v*) for 5 min. Noteworthy, the molar ratio of the template molecule (uric acid) and monomer (dopamine) was 1:10. The obtained Ni@BC-MIP films were characterized by such electrochemical methods: CV, EIS, and DPV. The rather long list of interfering substances makes this work exceptional. As possible interfering molecules in this study were analyzed: ascorbic acid, dopamine, glutamic acid, arginine, glucose, sucrose, adenine, hypoxanthine, xanthine, guanine, and allantoin. The obtained results demonstrated the linear range between current response and UA concentration was 0.01–30 μM. The study declares that uric acid recognition on Ni@BC-MIP depends on the hydrogen bond to maintain the template-polydopamine complex.

Some articles were addressed on the evaluation of poly(melamine–co-chloranil) imprinted with uric acid and deposited on the hanging mercury electrode [76,77] or on the graphite electrode [78]. The same scientific group described all three studies. There are several key points due to which the works distinguish from others. Differential pulse cathodic stripping voltammetry was used as a main electrochemical method for the qualification of the sensors. Hot water heated up to 80 °C was used for the extraction of the template molecule from the poly(melamine–co-chloranil) with uric acid imprints. The longest list of the interfering molecules was evaluated in the mentioned studies. The interfering molecules were selected according to their structural similarity, oxidation potential, and presence in the clinical samples: caffeine, theophylline, xanthine, hypoxanthine, allantoin, cytosine, glucose, thiourea, ascorbic acid, adenine, urea, histidine, uracil, and cytosine.

**Figure 3:**
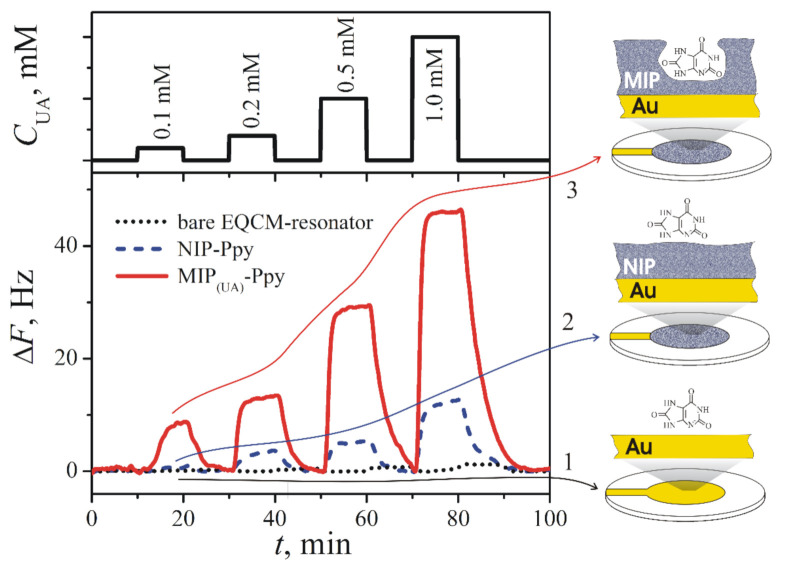
Changes of the resonance frequency (ΔF, Hz) of (1) bare gold-electrode-based (Au) EQCM-resonator (dotted line), (2) EQCM-resonator modified by NIP-Ppy (dashed line), and (3) EQCM-resonator modified by MIP(UA)-Ppy (solid line) after the addition of different concentrations of uric acid dissolved in 50 mM PBS, pH 7.0 [12]. Copyright 2021 by Elsevier. Reprinted with permission.

Uric acid-imprinted MIP based on Ppy was developed for measurements by EQCM [12]. Such Ppy-based MIP was deposited on gold electrode by the application of a single potential pulse of 1 V vs Ag/AgCl lasting for 10 s from solution containing a 5 mM of uric acid and 50 mM of pyrrole in PBS, pH 7.0. After polymerization, the template molecule was extracted from Ppy by continuous washing the 5 mL volume EQCM-cell at constant HPLC pump speed 1 mL/min with PBS for at least 30 min. Hence, for the extraction of uric acid from Ppy, the volume of PBS equal to 6 volumes of EQCM-cell was used. The registered results demonstrated linearity from 0.1 to 1.0 mM (Figure 3). Selectivity of the Ppy-based MIP was proofed against caffeine and glucose.

MIP-based sensors with uric acid imprints are summarized in Table 1. Special attention is paid for such points: (a) the description of the imprinted material and the type of electrode; (b) the method applied to form the molecularly imprinted polymers; (c) the application of extraction method of the template molecule from the MIP; (d) the choice of suitable methods for the characterization of formed MIPs; (e) and the selection of appropriate methods for electrochemical determination of the analytical signal; (f) the determination and identification of the interfering materials and application of proper strategies to reduce their influence and increase the performance of designed MIP-based analytical system.

**Figure 4:**
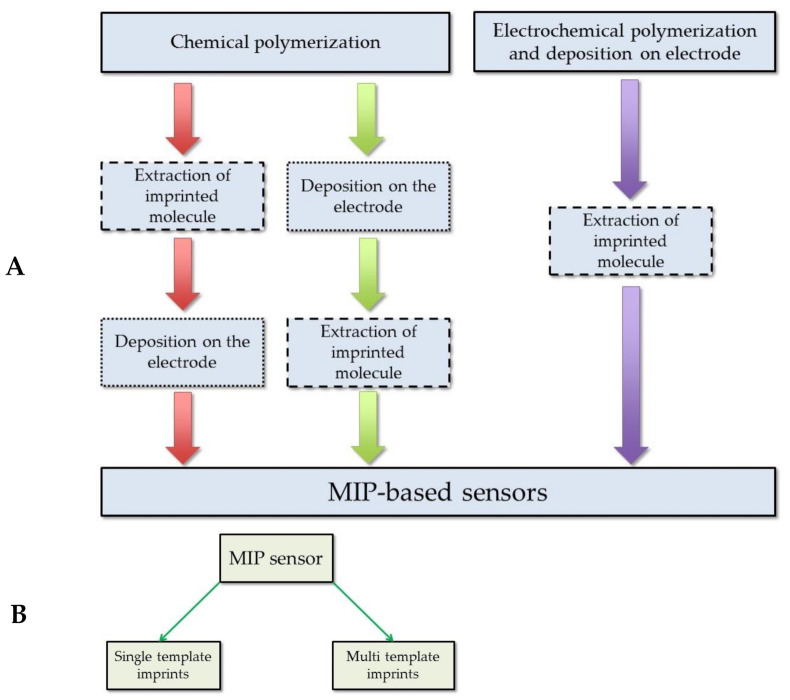
MIP-based sensor preparation protocol: (**A**) sensor preparation procedure; (**B**) the types of the sensors according to the number of imprinted molecules in the MIP.

The summarized protocol for MIP-based sensors preparation is depicted in Figure 4. The generalized procedure describes the principal of the MIP sensor preparation and discussed in the mentioned studies: the formation of the MIP with uric acid imprints by chemical or electrochemical methods, deposition on the electrode surface, and extraction of the imprinted molecule (Figure 4A). Two types of MIP sensors with the imprints of uric acid (Figure 4B) were described. The first type of MIP sensors is based on imprints of a single uric acid. Such type of MIPs is the most usually described in research. The second type of MIPs is based on the multi template imprints and they are dedicated for determination of uric acid simultaneously with other analytes [23,24,80]. The listed studies describe the MIT application to obtain the structures with imprints of uric acid. All these structures can be compared according to the criteria that are listed above (Table 1). It is obvious that the application of MIP allows a selective and sensitive determination of the target molecule in the presence of some interfering materials. As it was demonstrated in the research by Prasad research group [76,77,78], which reports the application of MIPs in qualitative and quantitative analysis for the determination of uric acid, these MIP-based structures enable to determine analytes in the samples that are containing many different interfering materials. The study segregates the results according to the structural similarity, oxidation potential, and presence in the clinical samples. Comparison of deposition methods used for the development of the uric acid sensors with MIPs allows drawing some observations about the simplicity of the procedure. The electrochemical deposition methods among all the methods used in studies that are listed in Table 1 can be remarked for their relative simplicity (Figure 4A). The application of electrochemical methods requires less optimization parameters and in the case of the deposition of MIPs by electrochemical methods such as potential cycling or constant potential-based deposition [12,14,23,24,26]. For potential cycling, the most critical are: (i) the number of cycles, (ii) potential sweep rate, and (iii) range between which potential is swept. For constant potential-based deposition, the most critical are: (i) applied potential and (ii) the duration of applied potential. During electrochemical deposition of MIPs, the concentrations of electro-polymerizable monomers and template molecules are very important, as well as the composition of solvent and pH. However, the last it was mentioned above electrochemical deposition mostly requires less optimization compared to other methods. Meanwhile, when using the chemical polymerization methods, the formed MIPs require an additional step to immobilize the MIP-based particles on the electrode surface. It means that the chemical polymerization method also needs to optimize the concentration of the solvent in which the monomers and the template molecule are dissolved, but at the same time, another criteria arises that determine the quality of the coating on the electrode. Such an aspect can be revealed in the study where the obtained MIP layers were spin coated over electrodes [78,80], or initially prepared carbon paste electrode were modified with MIPs [75], or when dipping the electrode into the MIP suspension or deposition of a certain amount of MIP suspension on the electrode surface [74,79]. Comparison of extraction methods of uric acid from the final structure of the MIP reveals that simple washing with water at elevated temperatures (ca. 80 °C) is sometimes sufficient to succeed sufficient extraction of the template from the MIP [76,78,79,81].

## 5. Conclusions

MIPs are offering many analytical and/or technological advantages for electrochemical and some other analytical systems dedicated to the determination of uric acid, which is an important issue in biomedicine, environmental and food chemistry. The possibility to design molecular imprints of uric acid within various polymers enables to design MIPs, which can replace enzymes mostly used for the determination of uric acid. Various polymers are suitable for the formation of MIPs, but among them, the most suitable are conducting polymers such as polypyrrole, which can be deposited electrochemically on conducting surfaces. In addition, Ppy can be easily overoxidized and by this procedure, selectivity of the formed MIP-layer can be remarkably increased. For this purpose, electrochemical MIP-formation methods are the most suitable and attractive because they enable to form conducting polymer layers in a controlled fashion and it is possible to adjust the most efficient electrodeposition conditions, which enables us to achieve rather good selectivity and sensitivity of MIP-based analytical systems.

## Figures and Tables

**Table 1:** Summary of MIP-based sensors with uric acid imprints: deposition methods, extraction of uric acid, the used evaluation methods, and interfering molecules.

Ref.	MIP or MIT, Electrode	Deposition Method	Extraction of Uric Acid	Evaluation Methods	Linear Range	LOD	Interfering Molecules
[24]	2-amino-5-mercapto-1, 3, 4-thiadiazole (AMT) with reduced graphene oxide on GCE	Electrochemical: CV	With the ethanol solution for 30 min	TEM, AFM, The selected-area electron diffraction (SAED), SEM, XPS, CV, EIS, and DPV in presence of redox probe.	0.01–100 μM	0.0032 μM	Dopamine, epinephrine, adenine, xanthine, ascorbic acid, and glucose.
[23]	o-phenylenediamine with nanoporous gold leaf on GCE	Electrochemical: CV	In 0.2 M H_2_SO_4_ by CV, 20 cycles from −0.5 V to + 0.5 V at a scan rate of 100 mV/s.	SEM CV, EIS, and DPV in presence of redox probe.	5.0–160 μM	0.4 μM	Urea, ascorbic acid, glucose, 3,4-dihydroxyphenylacetic acid (DOPAC), epinephrine, norepinephrine.
[51]	TiO_2_ on QCM electrode	NPs of TiO_2_ formed by sol-gel hydrolysis of Ti(O-nBu)_4_, on electrode transferred by dipping in TiO_2_ with MIT containing solution	Drying at 80 °C for 2 h and then calcination in air at 350 °C for 3 h	Piezoelectricity	0.04–45 μM	0.01 μM	Ascorbic acid, urea, glucose, glutamic acid, purine, and cytosine
[26]	Graphene doped chitosan on GCE	Electrochemical: chronoamperometry	By CV, 40 cycles from 1.5 V to −1.0 V at a scan rate of 0.8 V/s in 0.1 M PBS.	SEM, FTIR, CV, EIS, chronocoulometry, in presence of redox probe.	0.02–10.0 μM	-	Ascorbic acid, dopamine, and urea.
[74]	Poly-methacrylic acid (PMAA) on the surface of multi-walled carbon nanotubes (MWCNTs) on GCE	MIP particles formed by chemical polymerization, a certain amount of MIP particles dropped on electrode and evaporated the solvent	Methanol/water (3:1, *v*:*v*)	SEM, CV, linear sweep voltammetry, chronoamperometry.	80–500 μM	22 μM	Ascorbic acid.
[75]	Methacrylate, on carbon paste electrode	MIP particles formed by thermal polymerization, MIP particles mixed with graphite and eicosane to form a carbon paste	MIP particles washed for nine times using 50 mL, 0.1 N HCl and ethanol mixture (1:1 *v*/*v*) with stirring for 4 h.	FTIR, CV, differential pulse adsorptive stripping voltammetry (DPAdSV), EIS	0.5–100 μM	0.1 μM	Glucose, glycine, tryptophan, and ascorbic acid.
[14]	Carbon-entrapped nickel NPs (Ni@BC) coated with polydopamine, on GCE	Electrochemical: CV	The methanol/acetic acid solution (9:1, *v*/*v*) for 5 min.	XRD, SEM, TEM, XPS DPV	0.01–30 μM	0.008 μM	Ascorbic acid, dopamine, glutamic acid, arginine, glucose, sucrose, adenine, hypoxanthine, xanthine, guanine, and allantoin.
[76]	[poly(melamine-co-chloranil), on HMDE	Chemical polymerization of MIP, on HDME coated chronoamperometrically	Hot water, 80 °C	IR Differential pulse, cathodic stripping voltammetric (DPCSV)	0.015–2.75 μM	0.005 μM	Caffeine, theophylline, xanthine, hypoxanthine, allantoin, cytosine, glucose, thiourea, ascorbic acid, adenine, urea, histidine, uracil, and cytosine.
[77]	[poly(melamine-co-chloranil), on HMDE	Chemical polymerization of MIP, on HDME coated chronoamperometrically	Hot water, 80 °C	Differential pulse, cathodic stripping voltammetric (DPCSV), CV	0.65–23.8 μM	0.14 μM	Caffeine, theophylline, xanthine, hypoxanthine, allantoin, cytosine, glucose, thiourea, ascorbic acid, adenine, urea, histidine, uracil and cytosine.
[78]	poly(melamine-co-chloranil), brush grafted to tetraethoxysilane derived sol-gel thin film graphite electrode	Chemical polymerization of MIP; sol-gel of SiO_4_ in presence of MIP: spin coated on electrode	Hot water, 80 °C	SEM, IR, Differential pulse, cathodic stripping voltammetric (DPCSV).	87–1000 μM	24 μM	Caffeine, theophylline, xanthine, hypoxanthine, allantoin, cytosine, glucose, thiourea, ascorbic acid, adenine, urea, histidine, uracil, and cytosine.
[12]	Polypyrrole on EQCM electrode	Electrochemical: chronoamperometrically	PBS, for 30 min, at 1 mL/min of HPLC pump.	Piezoelectricity	0.1–1 mM	–	Caffeine, glucose.
[79]	Fe_3_O_4_@C modified with molecularly imprinted TiO_2_ on the magnetic GCE	Sol-gel hydrolysis TiO_2_ on electrode transferred by dipping in TiO_2_ with MIT containing solution	Multiple extractions (*n* = 8, extraction time 10 min) with 10 mL hot water (ca. 80 °C).	Photocurrent response, XRD, CV, TEM	0.3–34 μM	0.02 μM	Ascorbic acid, glutamic acid, cytosine, glucose, purine, and urea.
[80]	hyperbranched polymer (dendrimer) with dispersed GNPs-fMWCNTs on the PGE	Free radical polymerization of MIP particles, that were spin coated on the PGE	TEA-methanol (1:1, *v*/*v*)	SEM, DPASV	0.01–0.27 μM	0.0023 μM	Ascorbic acid, epinephrine, dopamine, L-tyrosine, L-tryptophan, creatinine, creatine, serotonine, glycine, glutamic acid, glucose, urea.
[81]	Imprinted zeotlite on GCE	Hydrothermal synthesis of zeolite. Zeolite on electrode transferred by potential cycling.	Warm water	XRD, FTIR, voltammetry	5.6–28 nM	5.9 nM	Ascorbic acid, creatine, and creatinine

GCE—glassy carbon electrode; GNPs-fMWCNTs—gold NPs functionalized multiwalled carbon nanotubes; HMDE—hanging mercury drop electrode; PGE—pencil graphite electrode; SEM—scanning electron microscopy.
